# Follicular Lymphoma

**DOI:** 10.4274/tjh.2014.0380

**Published:** 2015-02-15

**Authors:** İrfan Yavaşoğlu

**Affiliations:** 1 Adnan Menderes University Faculty of Medicine, Department of Hematology, Aydın, Turkey

**Keywords:** Follicular lymphoma, FLIPI, Rituximab

## TO THE EDITOR

The article entitled “Renal Infiltration of Follicular Lymphoma”, written by Petkovic et al. and published in one of the recent issues of your journal, was quite interesting [1]. Here we would like to emphasize some relevant points.

Extranodal disease is relatively common; any organ may be involved. The most common sites of extranodal disease include the bone marrow, skin, gastrointestinal tract, and bone [2]. Diagnosis of primary renal lymphoma based on the axillary involvement may not be accurate.

There are two Follicular Lymphoma International Prognostic Index (FLIPI) scores: FLIPI scores 1 and 2. FLIPI 1 consists of age, Ann Arbor stage, hemoglobin level, serum LDH level, and number of nodal sites. FLIPI 2 consists of β2 microglobulin, bone marrow involvement, hemoglobin level, largest diameter of lymph node, and age [3,4]. Treatment may be delayed if the following conditions are not present: B symptoms or pruritus, rapid generalized disease progression, marrow involvement, life-threatening organ involvement, renal infiltration, or bone lesions [5].

The results of the Primary Rituximab and Maintenance (PRIMA) study demonstrated an advantage in progression-free survival for rituximab maintenance therapy offered after initial chemoimmunotherapy [2]. The patient should receive maintenance rituximab treatment.
