# Supplementary material for: Follicular Lymphoma
Source: Turk J Haematol. 2015 Feb 15;32(1):94–5. doi: 10.4274/tjh.2014.0380 (PMC4439921; doi:10.4274/tjh.2014.0380)
Supplement: Related texts

# Reply: Authors' Response

**Authors:** Petkovic Ivan

## REPLY

Thank you very much for your kind invitation to reply to comments on our article entitled “Renal Infiltration of Follicular Lymphoma” which has been published in your respected journal in volume 31 issue 3 in 2014.

We presented a rare case of follicular lymphoma (FL) which was diagnosed after nephrectomy of the involved kidney which is well documented inhistopathology/immunochistochemistry pictures. The diagnosis was set up after CT scan which showed a renal mass mimicking renal cell carcinoma (RCC) but even so we avoided to use of the term primary renal lymphoma since we were not sure if it was FL which originated from the kidney or it was the infiltration of the kidney from the surrounding perirenal tissue. However, this is not relevant since it was extranodal FL for sure, which is quite rare to be found in the renal region of the body. After a surgery, performed CT scans of the body did not show the disease spreading so we decided to use a PET/CT scan, before making a decision on eventually “watch and wait” strategy. PET/CT scan found axillary lymph nodes to be positive with a high SUV (max 7). The situation became debatable since the grade of FL was 3A and SUV was high and we assumed that FL might be transforming in a more aggressive variant such as diffuse large B-cell lymphoma (DLBCL). Both FLIPI indexes were counted for the patient but we mentioned only FLIPI1 since there was a limited space for word counts for letters to the editor, so we did not have a place to comment on every aspect of analysis we have done. Our single institution decision was to treat the patient with R-CHOP. This can be debatable and we already commented in the text about our arguments to initiate induction therapy (grade 3 A and B can be treated like DLBCL as was appointed by one of the leading authorities in the field of FL treatment-Prof Ghielmini and there are publications that support the use of combined surgery+immunochemotherapy in renal lymphoma involvement but only if one kidney is involved [1], which was the situation with our patient). We already gave our arguments pro and contra the use of anthracyclines. The patient received 8 cycles and achieved a complete remission which was PET/CT verified.

If complete remission and long PFS is to be achieved, rituximab in combination with chemotherapy such as CHOP or bendamustine should be used [I, B] [2,3]. CVP combination results in inferior PFS, but no impact on OS was observed between these chemotherapy regimens [4].

Rituximab maintenance for 2 years improves PFS (75% versus 58% after 3 years, p<0.0001) [I, B] [5], whereas a shorter maintenance period results in inferior benefit [5,6]. Our patient underwent rituximab maintenance which still lasts; once again word counts were the major limiting factors to comment on every aspect of the treatment.

## References

[1] Cupisti A, Riccioni D, Carulli G, Paoletti S, Tognetti A, Meola M, Francesca F, Barsotti G, Petrini M (2004). Bilateral primary renal lymphoma treated by surgery and chemotherapy. Nephrol Dial Transplant.

[2] Rummel MJ, Niederle N, Maschmeyer G, Banat GA, von Grunhagen U, Losem C, Kofahl-Krause D, Heil G, Welslau M, Balser C, Kaiser U, Weidmann E, Durk H, Ballo H, Stauch H, Roller F, Barth J, Hoelzer D, Hinke A, Brugger W, Stauch H, Study group indolent Lymphomas StiL (2013). Bendamustine plus rituximab versus CHOP plus rituximab as first-line treatment for patients with indolent and mantlecell lymphoma: an open-label, multicentre, randomised, phase 3 non-inferiority trial. Lancet.

[3] Flinn IW, van der Jagt R, Kahl BS, Wood P, Hawkins TE, Macdonald D, Hertzberg M, Kwan YL, Simpson D, Craig M, Kolibaba K, Issa S, Clementi R, Hallman DM, Munteanu M, Chen L, Burke JM (2014). Randomized trial of bendamustine rituximab or R CHOP/R-CVP in first-line treatment of indolent NHL or MCL: the BRIGHT study. Blood.

[4] Federico M, Luminari S, Dondi A, Tucci A, Vitolo U, Rigacci L, Di Raimondo F, Carella AM, Pulsoni A, Merli F, Arcaini L, Angrilli F, Stelitano C, Gaidano G, Dell’olio M, Marcheselli L, Franco V, Galimberti S, Sacchi S, Brugiatelli M (2013). RR-CVP versus R-CHOP versus R-FM for the initial treatment of patients with advanced-stage follicular lymphoma: results of the FOLL05 trial conducted by the Fondazione Italiana Linfomi. J Clin Oncol.

[5] Salles GA, Seymour JF, Feugier P, Offner F, Lopez-Guillermo A, Belada D, Xerri L, Bouabdallah R, Catalano J, Pauline B, Caballero D, Haioun C, Pedersen LM, Delmer A, Simpson D, Leppa S, Soubeyran P, Hagenbeek A, Casanovas O, Intragumtornchai T, Ferme C, Gomes de Silva M, Sebban C, Lister A, Estell JA, Milone G, Sonet A, Coiffer B, Tilly H (2013). Updated 6 year follow-up of the PRIMA study confirms the benefit of 2-year rituximab maintenance in follicular lymphoma patients responding to frontline immunochemotherapy. Blood.

[6] Taverna CJ, Martinelli G, Hitz F, Mingrone W, Pabst T, Pabst L, del Giglio A, Vanazzi A, Laszlo D, Raats J, Rauch D, Vorobiof DA, Lohri A, Zucca E, Biaggi Rudolf C, Rondeau S, Rusterholz C, Ghielmini M (2013). Rituximab maintenance treatment for a maximum of 5 years in follicular lymphoma: results of the randomized phase III trial SAKK 35/03. ASH.
